# Poisoning by Coculus Indicus

**Published:** 1848

**Authors:** H. Rosenkrans


### ARTICLE V.

### *Poisoning by Coculus Indicus. By* H. Rosenkrans, M. D.

On Thursday last we witnessed in our town the effects of poisoning with Coculus Indicus on a man about forty years of age; he was a stranger who came to our town the day previous to taking the poison. Being an inebriate, he found and drank a liquid containing it, which had been left in a horse stable, where it had been prepared in whiskey to kill lice. It was early in the day that he took the fatal draught, and before he had taken anything in his stomach. After drinking the poison he felt immediately alarmed, and complained of sickness at the stomach, and had tremor; he then started from the stable, and, after walking a few rods, fell in the street, violently convulsed, and delirious, both during and after the paroxysm; a second and a third fit of convulsions, like the first, followed at sh.rt intervals. This last convulsive fit left him in a state of deep coma, in which condition he remained till death, which followed within an hour after the draught had been taken.

*Post Mortem.*---Stomach tinged yellow; mucous membrane slightly contracted; heart and lungs filled with dark fluid blood. The brain was not examined, yet, from the congested face and eyes and the deep coma, we had good reason to conclude that the brain was deeply congested. The examination took place five hours after death. A want of time prevented the inspection of the brain.

